# A Comparison of the Effects of Alpha and Medical-Grade Honey Ointments on Cutaneous Wound Healing in Rats

**DOI:** 10.1155/2016/9613908

**Published:** 2016-11-03

**Authors:** Shahram Paydar, Majid Akrami, Amirreza Dehghanian, Roshanak Alavi Moghadam, Mohsen Heidarpour, Amir Bahari Khoob, Behnam Dalfardi

**Affiliations:** ^1^Trauma Research Center, Shahid Rajaee (Emtiaz) Trauma Hospital, Shiraz University of Medical Sciences, Fars Province, Shiraz, Iran; ^2^Department of General Surgery, Shiraz University of Medical Sciences, Fars Province, Shiraz, Iran; ^3^Breast Diseases Research Center, Shiraz University of Medical Sciences, Fars Province, Shiraz, Iran; ^4^Department of Pathology, School of Medicine, Shiraz University of Medical Sciences, Fars Province, Shiraz, Iran; ^5^Student Research Committee, Shiraz University of Medical Sciences, Shiraz, Iran; ^6^Department of Internal Medicine, Shiraz University of Medical Sciences, Shiraz, Iran

## Abstract

*Introduction*. This study compared the healing efficacy and possible adverse effects of topical Alpha and medical-grade honey ointments on cutaneous wounds in rats.* Methods*. To conduct the study, 22 male Sprague-Dawley rats were randomly allocated into two equal groups: (1) rats with Alpha ointment applied to the wound surface area and (2) rats with medical-grade honey ointment applied to their wounds. The ointments were applied daily during the 21-day study period. Wound contraction was examined photographically with images taken on days 0, 7, and 21 after wounding. The healing process was histopathologically assessed using skin biopsies taken from the wound sites on days 7 and 21.* Results*. No statistically significant difference in mean wound surface area was observed between the two study groups. According to histopathological assessment, a significant reduction in the amount of collagen deposition (*P* value: 0.007) and neovascularisation (*P* value: 0.002) was seen in the Alpha-treated rats on day 21. No tissue necrosis occurred following the application of Alpha ointment.* Conclusion*. Daily topical usage of Alpha ointment on a skin wound can negatively affect the healing process by inhibiting neovascularization. Topical Alpha ointment can reduce the possibility of excessive scar formation by reducing collagen deposition.

## 1. Introduction

Wounds, especially chronic ones, are now a major health concern affecting a large number of patients and causing a considerable reduction in their health-related quality of life [1, 2]. For this reason, research on wound healing agents is currently an attractive and developing field in biomedical sciences [2, 3].

One group of drugs that has shown significant capabilities in the area of wound care and management is herbal medicines [3]. The use of plant materials has been a common medical practice since early times, especially in eastern countries; even now, various wound care products contain herbal ingredients [2, 3]. One such herbal medicine presently available in Iran and claimed to improve wound healing is Alpha ointment [4, 5].

Alpha ointment contains the active ingredient Lawsone (available in natural henna (*Lawsonia inermis* Linn.)) and unsaturated fatty acids [4–6]. According to previous research, this composition has antioxidant and anti-inflammatory features [5, 6]. It has also been claimed that Alpha ointment is beneficial in improving wound healing [4]. As we see in our daily clinical practice, Alpha ointment is one of the prescriptions commonly used for the management of lower extremity chronic wounds, particularly diabetic ulcers. However, data regarding its efficacy in wound management is insufficient, and there are no strong and well-documented recommendations for its use. Based on these facts, the current experimental study evaluated the ability of Alpha ointment to improve skin wound healing and compared it with the effects of medical-grade honey ointment, a product that received U.S. Food and Drug Administration (FDA) approval for use in conditions like leg ulcers, burns, diabetic foot ulcers, traumatic wounds, and so forth [7].

## 2. Materials and Methods

### 2.1. Ethical Approval

The study protocol was approved by the Animal Ethics Committee of Shiraz University of Medical Sciences, Shiraz, Iran. All procedures were performed under general anesthesia, and all efforts were made to minimize the animals' suffering.

### 2.2. Animals and Excisional Wound Model

In this study, we used a previously examined method [9]. A total of 22 healthy adult male Sprague-Dawley rats (mean weight: 350 g) were selected for this experimental study. The rats were kept in separate clean cages and had free access to equal amounts of standard food (Center of Comparative and Experimental Medicine, Shiraz University of Medical Sciences, Shiraz, Iran) and water. They were housed in temperature-controlled (22 ± 2°C) and humidity-controlled (55 ± 15%) rooms with 12-hour light/dark photoperiods and allowed to adapt to their environment for one week before experiments began.

The rats were randomly and equally allocated into two groups (*n* = 11): (1) rats for which Alpha ointment (Rejuderm, Iran) was applied to the wound surface area (Alpha-treated group) and (2) rats for which medical-grade honey ointment (Medihoney®, Comvita Ltd., New Zealand) was applied to the wounds (honey-treated group). During the 21-day study period, the ointments were applied to the wound surface areas at 24-hour intervals with disposable applicators in a manner that created a thin layer that fully covered the wound.

To generate the wounds, the rats were first anesthetized with an intramuscular injection of thiopental sodium (40 mg/kg; Biochemie, GmbH, Austria) and xylazine (10 mg/kg; Alfasan International, Woerden, Netherlands). Then, their back hair was shaved, and the wound site was disinfected using alcohol ethylic solution. Next, a full-thickness circular excisional skin wound (20 mm in diameter and 2 mm deep) was created on the back of each rat using scissors and forceps.

Throughout the study period, the rats' wounds were carefully examined every day for any possible complications, particularly any macroscopical manifestation of infection. Of note, rats were to be excluded from the experiment if death occurred.

### 2.3. Photographical Evaluation of Wound Healing

Wound contraction was assessed photographically with images taken using a digital camera (PowerShot G9 12.1 Megapixel Camera; Canon, Tokyo, Japan) on days 0, 7, and 21 after wounding. The camera was fixed at a distance of 10 cm from the wound surface (in a vertical view), and a fine-line ruler was held at wound level at the time of photography in order to calibrate the magnification of the photographs. Photos were analyzed using Adobe Photoshop CS program (Adobe Systems, San Jose, CA, USA) (analysis menu > record measurements command).

### 2.4. Histopathological Evaluation of Wound Healing

Semicircular full-thickness skin biopsies from wound sites in both groups were taken on days 7 (half of the wound site with a margin of 2 mm) and 21 (the remaining part with a 2 mm margin) after wounding. Animals were first anesthetized with inhaled* ether* on day 7 and then euthanized with* ether* on day 21.

After the biopsy, specimens were washed with sterile normal saline. Tissue samples were immediately fixed in buffered formaldehyde (10% formalin) and then sent for histopathological assessments (haematoxylin and eosin and Masson-trichrome staining and light microscopic evaluation).

The scoring system described by Abramov et al. for the histopathological evaluation of physiological parameters involved in the wound healing process was adapted for use in this study (Table 1) [8]. Abramov's scoring system examines the following criteria: amount of acute and chronic inflammatory infiltrates, amount of granulation tissue, maturation of granulation tissue, collagen deposition, neovascularization, and reepithelialization. All investigators who assessed tissue samples or analyzed images in this study were blinded to the agents given.

### 2.5. Statistical Analysis

The results are presented as mean ± standard deviation (SD). Statistical comparisons were made using the Mann–Whitney* U *test (SPSS Statistics software, version 16; Chicago, Illinois, USA). A *P* value less than 0.05 was considered significant.

## 3. Results

### 3.1. Wound Contraction

The mean ± SD values of wound surface area were calculated for each group using images taken on days 0, 7, and 21 after wounding (Table 2). The results indicated no statistically significant difference between the two groups on the aforementioned study days. Of note, all rats survived the experiment, and their wounds showed no apparent signs of infection during the study period.

### 3.2. Histopathological Examinations

**Figure 1:**
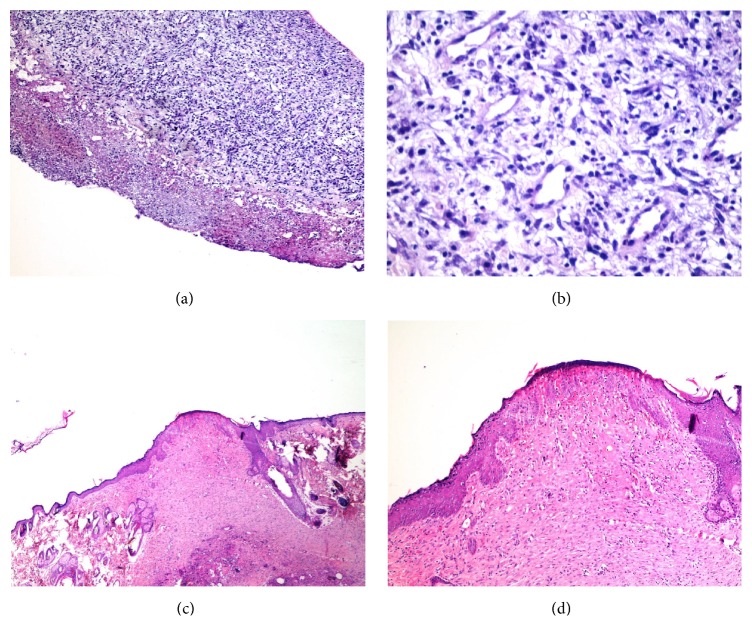
Histopathologic changes of ulcers in Alpha-treated group on day 7 showed ulceration, chronic inflammation, and granulation tissue formation: (a) ×400 and (b) ×400. Histopathologic changes in lesions of the same group on day 21 showed full reepithelialization and neovascularization: (c) ×40 and (d) ×100.

**Figure 2:**
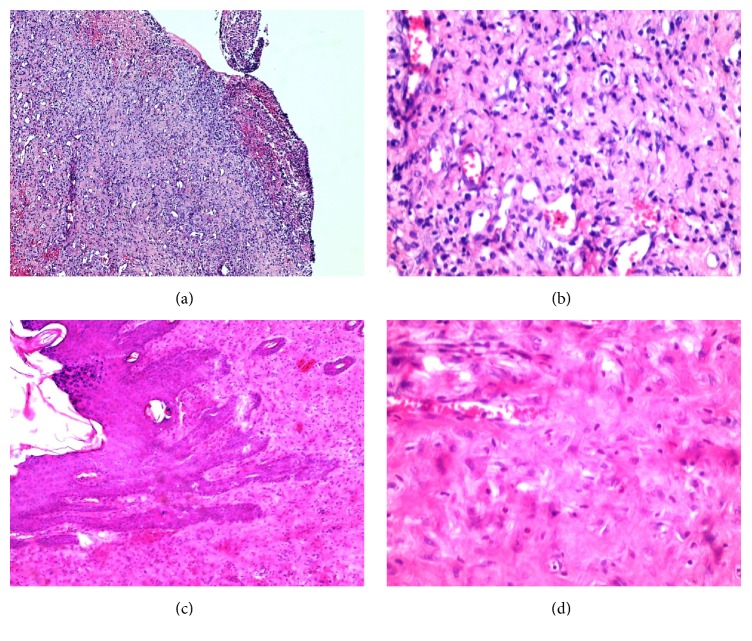
Histopathologic changes in the ulcers of honey-treated group on day 7 showed ulceration, chronic inflammation, and granulation tissue formation: (a) ×40 and (b) ×400. Histopathologic changes in the lesions of the same group on day 21 showed full reepithelialization, subepidermal fibrosis, and scar formation: (c) ×100 and (d) ×400.

The results of the histopathological assessment (Figures 1 and 2) are summarized in Table 3. According to the findings, there was no statistically significant difference between the Alpha- and honey-treated groups regarding the factors of acute and chronic inflammation, amount and maturation of granulation tissue, and reepithelialization on days 7 and 21. However, significant differences in collagen deposition (*P* value: 0.007) and neovascularisation (*P* value: 0.002) between the two groups were observed on day 21. The histopathological examination of the skin biopsies revealed no tissue necrosis following the application of Alpha ointment.

## 4. Discussion

Finding new agents to accelerate wound healing and improve currently available products is the main concern for researchers in biomedical sciences [10]. Herbal medicines are a favourite agent used for this purpose [3]. One such agent, Alpha ointment containing Lawsone from henna and some other ingredients, has been produced in Iran in recent years [4–6]. Issues concerning herbal-derived wound care products are their efficacy and possible adverse dermatological effects associated with their use [11]. Because of these issues and the fact that documented evidence about Alpha ointment is insufficient, this study examined the effects of this product on the parameters involved in the skin healing process and compared it with the FDA-approved product medical-grade honey ointment [7].

Some previous studies defend the antibacterial properties of* Lawsonia inermis* and its efficacy in acceleration of wound repair [12–14]. The experiment of Shivananda Nayak et al., who conducted an animal study to evaluate the impact of ethanol extract of* Lawsonia inermis* (the plant on which Alpha ointment is based), is an example for this case [12]. According to their results, this agent had the ability to increase the rate of wound contraction, decrease the period of epithelialization, and significantly increase the granulation tissue weight when compared with controls [12]. These authors' histological findings from the extract-treated group showed an increased amount of well-organized collagen bands (consequently the increased skin-breaking strength), increased number of fibroblasts, and reduced number of inflammatory cells compared with the controls [12].

However, few studies have directly assessed the efficacy of Alpha ointment for wound healing [4–6]. Ansari et al., using their study criteria, compared the efficacy of topical Alpha ointment with that of topical hydrocortisone (1%) in healing radiation-induced dermatitis in cases of breast cancer [6]. After following up the patients for three weeks, the authors concluded that the topical application of Alpha ointment was more effective in healing radiation-induced dermatitis than topical hydrocortisone cream (1%) [6]. Hosseini et al. compared the impact of Alpha ointment with that of silver sulfadiazine on the healing process of standard third-degree and* Pseudomonas aeruginosa* infected burn wounds [4]. According to their results, Alpha ointment significantly decreased the rate of wound infection, positive cultures, and scar formation compared with the other groups [4]. The authors reported that another notable advantage of Alpha ointment compared with some other products used in burn wound care, like silver sulfadiazine, was its lower price [4]. Moreover, they noted that Alpha ointment had very few and acceptable adverse reactions [4].

In the current study, significant differences were observed between Alpha- and honey-treated groups regarding the two factors of collagen deposition and neovascularisation on day 21. In contrast to Shivananda Nayak et al.'s work, this study revealed significantly lesser collagen deposition in the Alpha-treated group than in the honey-treated one [12]. This phenomenon reduces the possibility of excessive scar formation after the wound healing process (similar to what was seen in Hosseini et al.'s work) [4, 15–17]. Due to the role of collagen matrix and its organization in tissue tensile strength, the other probable aspect of reduced collagen deposition is its impact on the strength of the final healed tissue [18, 19]. However, a prerequisite for commenting about the significance of this event is examining it using standard methods and devices, a matter that was not evaluated in this work.

Another important point is the angioinhibitory property of Alpha ointment. It is evident that neovascularization (neoangiogenesis) is critical during wound healing to provide tissues with essential materials [20, 21]. Therefore, the inhibition of such event could negatively affect the healing process. However, in spite of the angioinhibitory function of Alpha ointment, photographical examinations in the current study revealed no statistically significant difference between the two examined groups regarding wound contraction and wound surface areas on the specified days.

This study had some limitations that need to be mentioned. First of all, for each rat, biopsies were obtained from the same healing wound on days 7 and 21 (half of the wound area was biopsied in each day), and this restricted the interpretation of wound surface area on day 21. Another limitation of this work was the limited number of skin biopsies obtained from each rat. In fact, to better monitor the healing process and better recognize possible differences, additional biopsies should have been taken early after wounding (e.g., on day 4) and between days 7 and 21.

## 5. Conclusion

According to this histopathological study, daily topical usage of Alpha ointment on a skin wound in rats can negatively affect the healing process by inhibiting neovascularization. Furthermore, because of the reduced collagen deposition within the healing tissues, there is lesser possibility of excessive scar formation after the topical application of Alpha ointment. However, further human* in vivo* studies are recommended to examine the clinical importance of these findings.

## Figures and Tables

**Table 1:** The histopathological scoring system for evaluation of wound healing [8].

Parameter	Score			
	0	1	2	3
Acute and chronic inflammation	None	Scant	Moderate	Abundant
Amount of granulation tissue	None	Scant	Moderate	Abundant
Granulation tissue maturation	Immature	Mild maturation	Moderate maturation	Fully mature
Amount of collagen deposition	None	Scant	Moderate	Abundant
Reepithelialization	None	Partial	Complete but immature or thin	Complete and mature
Neovascularization	None	Up to five vessels per HPF^*∗*^	6 to 10 vessels per HPF	More than 10 vessels per HPF

^*∗*^HPF: microscopic high power field.

**Table 2:** Mean ± SD of wound surface area (mm^2^) in Alpha- and honey-treated groups on different days after wounding.

Day	0	7	21
Alpha-treated	318.16 ± 13.22	46.54 ± 11.34	0.0 ± 0.00
Honey-treated	316.87 ± 13.67	50.83 ± 9.49	0.26 ± 0.49
*P* value	0.177	0.387	0.347

**Table 3:** Mean ± SD values of histopathological scores of wound healing among different study groups.

Parameter	Groups					
	Day 7		*P* value	Day 21		*P* value
	Alpha-treated	Honey-treated		Alpha-treated	Honey-treated	
Acute and chronic inflammation	1.27 ± 0.46	1.90 ± 0.94	0.15	1.36 ± 0.67	1.81 ± 0.87	0.24
Amount of granulation tissue	2.54 ± 0.52	2.54 ± 0.52	1.00	0.81 ± 0.87	0.81 ± 0.75	0.89
Granulation tissue maturation	2.45 ± 0.52	2.63 ± 0.50	0.47	1.63 ± 1.36	2.09 ± 1.37	0.36
Amount of collagen deposition	1.72 ± 0.64	2.00 ± 0.44	0.33	0.90 ± 0.70	2.00 ± 0.89	0.007^*∗*^
Reepithelialization	0.63 ± 1.02	0.54 ± 1.03	0.79	2.45 ± 0.93	2.72 ± 0.64	0.51
Neovascularization	3.00 ± 00	3.00 ± 00	1.00	2.18 ± 0.60	3.00 ± 0.00	0.002^*∗*^

*∗* indicates significant differences (*P* < 0.05).
